# Challenges and Controversies in Defining Totally Drug-Resistant Tuberculosis

**DOI:** 10.3201/eid1811.120526

**Published:** 2012-11

**Authors:** Peter Cegielski, Paul Nunn, Ekaterina V. Kurbatova, Karin Weyer, Tracy L. Dalton, Douglas F. Wares, Michael F. Iademarco, Kenneth G. Castro, Mario Raviglione

**Affiliations:** Author affiliations: Centers for Disease Control and Prevention, Atlanta, Georgia, USA (P. Cegielski, E.V. Kurbatova, T.L. Dalton, M.F. Iademarco, K.G. Castro);; World Health Organization, Geneva, Switzerland (P. Nunn, K. Weyer, D.F. Wares, M. Raviglione)

**Keywords:** tuberculosis, mycobacteria, Mycobacterium tuberculosis, multidrug-resistant, extensively drug-resistant, totally drug-resistant, drug resistance, bacteria

## Abstract

In March 2012, in response to reports of tuberculosis (TB) resistant to all anti-TB drugs, the World Health Organization convened an expert consultation that identified issues to be resolved before defining a new category of highly drug-resistant TB. Proposed definitions are ambiguous, and extensive drug resistance is encompassed by the already defined extensively drug-resistant (XDR) TB. There is no evidence that proposed totally resistant TB differs from strains encompassed by XDR TB. Susceptibility tests for several drugs are poorly reproducible. Few laboratories can test all drugs, and there is no consensus list of all anti-TB drugs. Many drugs are used off-label for highly drug resistant TB, and new drugs formulated to combat resistant strains would render the proposed category obsolete. Labeling TB strains as totally drug resistant might lead providers to think infected patients are untreatable. These challenges must be addressed before defining a new category for highly drug-resistant TB.

A 2011 report from India described 4 patients with tuberculosis (TB) for whom drug susceptibility testing (DST) of *Mycobacterium tuberculosis* cultures showed phenotypic resistance to all of 12 drugs examined (*1*). The authors of that report proposed the term: totally drug-resistant TB, abbreviated as TDR TB. To our knowledge, this was the fourth reported series of TB cases that showed resistance to all drugs tested, heralding the advent of TB strains resistant to all available anti-TB drugs (*1*–*5*).

In 2007, Migliori et al. (*2*,*3*) reported 2 cases in Italy and suggested use of the term extremely drug-resistant TB, which they abbreviated as XXDR TB. In 2009, Velyati et al. (*4*) reported 15 cases from Iran and suggested use of the terms, totally drug-resistant TB and super extensively drug-resistant (XDR) TB. Other reports have used different definitions of totally drug-resistant TB and terms such as super resistant and pre-XDR to describe different degrees of drug resistance, but with imprecise definitions (*6*,*7*). In the fourth report, Shah et al. (*5*) reported 13 cases in South Africa that exhibited resistance to all of the drugs that were available for treatment. Knowing that these cases were encompassed by the accepted definition for XDR TB (*8*), Shah et al. judiciously refrained from proposing more new nomenclature. The concept of resistance to all standard anti-TB drugs is easily understood in colloquial terms, but there is no precise consensus definition for degrees of drug resistance that are worse than that of XDR TB. Defining total drug resistance is challenging and controversial. However, lessons can be learned from our experience during 2005 and 2006, when XDR TB was defined.

In September 2005 and March 2006, the Centers for Disease Control and Prevention published data documenting the worldwide emergence of XDR TB, defined provisionally as a subset of multidrug-resistant TB (i.e., resistance to at least isoniazid and rifampin) that had additional resistance to at least 3 second-line anti-TB drugs (*9*,*10*). We chose this definition on the basis of data from 14 Supranational TB Reference Laboratories representing 18,225 TB patients (tested during 2000–2004) in 48 countries because, at the time, even this elite tier of mycobacteriology laboratories rarely tested >3 second-line drugs and because this degree of drug resistance functionally precluded adequate treatment with at least 4 otherwise effective drugs (*11*).

Subsequently, an emergency task force convened during 2006 by the World Health Organization (WHO) revised the definition of XDR TB because susceptibility testing for many anti-TB drugs was problematic, especially for ethambutol, pyrazinamide, the thioamides (ethionamide and prothionamide), the serine derivatives (cycloserine and terizidone), and para-aminosalicylic acid (*8**,**12**,**13*). The intrinsic reproducibility of DST for these drugs ranges from 50%–80% (*12*,*13*). The revised definition included resistance to any drugs of the fluoroquinolone class and 1 or more of the 3 second-line injectable drugs (kanamycin, amikacin, or capreomycin) in addition to isoniazid and rifampin (*8*). This definition was appealing for 3 reasons. First, it paralleled the definition of MDR TB. The definition of MDR TB included resistance to the 2 most effective first-line drugs. The revised definition of XDR TB focused on the 2 most effective classes of second-line drugs. Second, it depended on DST methods considered by leading mycobacteriologists to be reliable (>90% reproducibility). Third, in the short term, it supported more accurate case detection and surveillance, limiting under-diagnosis and under-reporting because DST was not available for at least 3 second-line drugs. Subsequent investigations confirmed that patients with XDR TB had substantially higher frequency of unfavorable outcomes than patients with MDR TB (*14*–*16*). The acronyms MDR and XDR were established largely on practical grounds, serving patients, providers, and programs. They have been sufficient for complex public health surveillance, treatment recommendations, and research purposes. In contrast, developing a definition for totally drug-resistant TB is problematic for several reasons. We list 7 challenges that should be addressed before new terminology should be considered for adoption.

The first challenge is that the definition should not hinge on resistance to all drugs tested, because the number of drugs tested varies widely between laboratories. Anti-TB drugs are now divided into 11 categories, some of which represent classes of closely related drugs on the basis of chemical similarity and frequency of cross-resistance: 1) isoniazid, 2) rifamycins (e.g., rifampin, rifabutin, rifapentine), 3) pyrazinamide, 4) ethambutol, 5) streptomycin, 6) second-line aminoglycosides (e.g., kanamycin, amikacin), 7) cyclic polypeptides (e.g., capreomycin, viomycin), 8) fluoroquinolones (e.g., ciprofloxacin, ofloxacin, levofloxacin, and moxifloxacin), 9) thioamides (e.g., ethionamide, prothionamide), 10) serine derivatives (e.g., cycloserine, terizidone), and 11) para-aminosalicylic acid.

It would be problematic for the proposed terminology if laboratories tested for 1 representative drug within each class of second-line drugs but not for all of the drugs in that class. For example, many laboratories test for resistance to kanamycin and ofloxacin but not for resistance to amikacin, capreomycin, levofloxacin, and moxifloxacin. Before strains can be said to be totally drug resistant or not totally drug resistant, laboratories would have to test for resistance to all of these drugs as well as the thioamides, serine derivatives, and para-aminosalicylic acid.

Along the same lines, the second challenge is that in vitro testing data suggest cross-resistance among different drugs within a class of drugs (e.g., the fluoroquinolones) or closely related classes of drugs (e.g., the aminoglycosides and polypeptides) is not 100%. For example, not all rifampin-resistant isolates are resistant to rifabutin, not all kanamycin-resistant isolates are resistant to amikacin and capreomycin, and not all ofloxacin-resistant isolates are resistant to moxifloxacin (*17*,*18*). Many isolates with resistance to low levels of isoniazid are susceptible to higher concentrations of isoniazid (*17*). Molecular analysis of the mechanisms of drug action and drug resistance has explained some of these differences, but this information is incomplete, and conventional growth-based DST and molecular tests are imperfect (*19*–*21*). Molecular detection of drug resistance is not always concordant with conventional growth-based detection by DST (*19*). Our increasing understanding of the biologic mechanisms for resistance is shedding light on some of these inconsistencies (*21*). Whether these subtle differences in bacterial resistance to different drugs within the same chemical class of drugs have any clinical impact is unknown and exceedingly difficult to study (*21*). If an isolate is resistant to ofloxacin but susceptible to moxifloxacin, would it count as totally drug resistant? The same question could be applied to kanamycin, amikacin, and capreomycin. These are among the reasons that the WHO/Centers for Disease Control and Prevention definition of XDR TB is based on resistance to drugs within a class of closely related drugs, i.e., any fluoroquinolone and any of the 3 second-line injectable drugs.

Given the global emergence of XDR TB, the third challenge is that physicians and patients have reached for so-called third-line drugs (*22*), some of which are also classified by WHO as Group 5 drugs (*23*), because of their in vitro effect against *M. tuberculosis* or because of their activity against other species of mycobacteria. These third-line drugs include amoxicillin/clavulanic acid, clofazimine, linezolid, metronidazole and other imidazoles, thioridazine and other phenothiazines, macrolides, and monobactams (imipenem, meropenem) (*24*–*28*). Clinical trials of some of these drugs (linezolid, clofazimine, metronidazole, clarithromycin, azithromycin) have been undertaken to define their effectiveness against drug-susceptible and drug-resistant disease (www.clinicaltrials.gov/ct2/search). Most mycobacteriology laboratories do not test *M. tuberculosis* for resistance to these drugs, and DST methods have not been standardized or widely accepted. Nonetheless, reference and research laboratories in several countries test for resistance to these drugs (*24*,*25*,*29*,*30*). If an isolate from a patient retains susceptibility to >1 of these drugs, it could not really be considered totally drug resistant. A good definition should address this issue.

The fourth challenge is that DST for several anti-TB drugs is not sufficiently reproducible or reliable; retesting the same isolate gives a different result in many cases (*20*). Moreover, different laboratories use different methods, and DST technology continues to evolve (*17*,*23*,*31*–*35*). Subcategorization of resistance beyond XDR enables this variability to become more of a confounding factor for surveillance and patient care. The reproducibility of DST for ethambutol, pyrazinamide, and ethionamide is ≈80% and the reproducibility of DST for para-aminosalicylic acid and cycloserine is ≈50%–60% (*12*,*13*,*36*). Since the original studies of the 1960s (*31**,**32*), few studies have compared the results of DST from different testing methods with the outcomes of patient care and treatment (*21*,*37*).

The fifth challenge is that there are several new anti-TB drugs under development that will be prototypes for new classes of antimicrobial drugs or add new chemical entities to existing classes. For example, the drugs bedaquiline (TMC207), delaminid (OPC67683), SQ109, PA824, AZD5847, and PNU100480 have entered human trials and may be available for clinical use within the next several years. Information about clinical trials with these drugs is available (http://www.newtbdrugs.org/pipeline.php), and promising results of phase II trials of bedaquiline and delaminid have been published (*38*,*39*). New DST methods and resistance standards must be developed for new drugs. An approach to defining total drug resistance should take into account these imminent events; otherwise, the definition will be outdated quickly.

The sixth challenge is that we must avoid the unintended implication that patients with total drug resistant TB should not or cannot be treated. These patients deserve consideration of compassionate access to promising new drugs and may require the off-label use of medications that have demonstrated in vitro action against *M. tuberculosis.*

The seventh and last challenge is that global laboratory capacity for DST of *M. tuberculosis* isolates remains limited, although all 29 Supranational TB Reference Laboratories now routinely perform DST to detect MDR TB and XDR TB. In the original survey of 14 Supranational TB Reference Laboratories noted, 7 laboratories had the capacity to test >11 classes of drugs, but only 2 of the 7 laboratories regularly tested all 11 drugs or classes of drugs on a regular basis (*11*). MDR TB was identified in 3,662 isolates; 234 (6.8%) of those were further classified as XDR TB. In the 7 laboratories with the capacity to test >11 classes of drugs, 11 classes were tested against 1,585 (43%) of the MDR TB isolates; 3 isolates (0.2% of MDR and 1.9% of XDR isolates) were resistant to the 11 classes tested. 

Studies have suggested that TB with resistance to all drugs tested is rare (*36*,*40*). Two laboratories tested all 11 classes of drugs in the case-based study of the DOTS-Plus pilot projects, which was a retrospective cohort of 1,768 case-patients with confirmed MDR TB in 5 countries from the first 5 DOTS-Plus pilot projects approved by the Green Light Committee of the Working Group on MDR TB of the Stop TB Partnership (36,*40*). Among 57 XDR TB case-patients (3.8% of 1,488 MDR TB cases) in these studies, 29 (51%) were tested for susceptibility to the11 main classes of drugs, and none were resistant to all classes. In the Preserving Effective TB Treatment Study, an ongoing prospective study of MDR TB cases in 9 countries, 87 (6.6%) of 1,328 MDR TB isolates were identified as XDR TB (*18*). Of these, 1 had resistance to all 12 drugs tested at the start of treatment. The strengths of the methods of that study were that susceptibility testing was centralized and all isolates were tested against the same 12 drugs.

These 2 studies and the report by Shah et al. from South Africa (*5*) highlight the issue of different circumstances under which drugs are tested in relation to drug availability in different countries. Depending on the availability of drugs in a specific country, it might not matter if resistance to 11 categories of drugs is detected. If a strain is resistant to all of the drugs available, then it would be functionally equivalent to total drug resistance from the perspective of the patient and the health care provider.

To address these complex issues, WHO convened an expert consultation on the nosology of drug-resistant TB in March 2012, adjacent to an expert consultation on developments in anti-TB DST, to review systematically the available evidence bearing on this matter (www.who.int/tb/challenges/xdr/xdrconsultation/en/index.html). In summary, existing guidelines cover the range of highly drug-resistant forms of TB, including case definitions and treatment recommendations (*8*,*22*,*23*). The 2006 definition of XDR TB encompasses additional drug resistance (*8*,*23*). Available data do not support the need to establish this new category on the basis of substantial differences from XDR TB, apart from the potential impact of such new nomenclature on public awareness and advocacy for increasing resources for TB control.

Proper evaluation of yet another definition must rigorously consider these challenges and controversies with the aim of improving clinical care and public health. Essential criteria for such a definition should include 1) the specific drugs in the definition, 2) consideration of third-line drugs and new drugs that will be available shortly, 3) reproducibility of the DST methods used to compose the definition, and 4) a clear difference in treatment recommendations and treatment outcomes between patients who would be included in any new definition and those who can be classified for recording and reporting purposes on the basis of current definitions. As countries increase treatment of MDR and XDR TB, it is inevitable that resistance to second-line drugs will increase. New drugs and better diagnostic tools are needed urgently for patients with highly drug-resistant TB.
